# Regarding Case Report "Povidone Iodine Irrigation - A Possible Alternative to Lead Extraction"

**DOI:** 10.1016/s0972-6292(16)30570-8

**Published:** 2012-12-02

**Authors:** Akbar Shafiee, Ali Kazemisaeed

**Affiliations:** 1 Research assistant, Department of cardiovascular research, Tehran Heart Center, Tehran University of Medical Sciences; 2 Associate professor, Department of electrophysiology, Tehran Heart Center, Tehran University of Medical Sciences

**Keywords:** Pacemaker, infection, conservative treatment

## Abstract

We read the case report of Dr. Puri and colleagues for treating an infected permanent pacemaker conservatively in an elderly patient. Due to its similarity of this work with ours, we wish to highlight some points regarding conservative treatment of pacemaker infection.

We admire the courage of Dr. Puri and colleagues for treating an infected permanent pacemaker conservatively in an elderly patient [1]. This work was very similar to what we did and reported in 2010 [2]. However, our case was more complicated and the total treatment process continued for about 2 months. Above all, the pacemaker was kept out of its pocket for about 5 weeks, which is indeed a unique operation.

Given that both cases aim to introduce a conservative treatment method for pacemaker infection and as far as management of cardiac devices has always been a challenge, we wish to highlight the following points:

1. Pacemaker infections are diagnosed frequently, and there are even a number of patients subjected to several surgeries for pacemaker infections [3]. The challenging treatment is lead extraction and device removal, which imposes a considerable financial burden on the healthcare system and the patients themselves, not to mention the health issues.

2. Conservative treatment of pacemaker infection must be reserved for patients who are critically dependant on pacing and lead extraction may predispose this group of patients to unpredictable events and mortality. Ethical issues are extremely important in such decisions. Moreover, conservative treatment must be performed in a highly qualified center where all necessary facilities are available, especially in emergency situations.

3. Selection of the proper antiseptic for the sterilization of the wound and devices is also of significance. In our study, we used Decocept™, which is a commercial antiseptic, the ingredients of which are Isopropanol, N-Propanol and Chloride Benzalkonium. Nonetheless, the irritating effects of the Povidone Iodine are well documented [4]. This characteristic can delay wound healing and cicatrisation, thereby prolonging of the treatment period. We believe that the selection of the proper antiseptic should be individualized in every medical center, considering the allergic history of the patient, bacterial resistance profile and condition of the wound.

We hope that the abovementioned remarks can assist other clinicians in the future with their selection a conservative treatment modality in pacemaker infections.
